# Altered HIF-1α, Netrin-1, and Netrin-4 Levels in Obstructive Sleep Apnea: Associations with Intermittent Hypoxia and Disease Severity

**DOI:** 10.3390/medicina62071340

**Published:** 2026-07-11

**Authors:** Mehmet Erdem, Tuğba Raika Kıran, Nurcan Kırıcı Berber, Lale Şahin Gür, Şeniz Erdem

**Affiliations:** 1 Department of Medical Biochemistry, Faculty of Medicine, Malatya Turgut Özal University, Malatya 44210, Türkiye; 2 Department of Pulmonary Medicine, Faculty of Medicine, Malatya Turgut Özal University, Malatya 44210, Türkiye; 3 Department of Medical Biochemistry, Faculty of Medicine, Karadeniz Technical University, Trabzon 61080, Türkiye

**Keywords:** HIF-1α, hypoxic burden, intermittent hypoxia, Netrin-1, Netrin-4, obstructive sleep apnea

## Abstract

***Background and Objectives*:** Obstructive sleep apnea (OSA) is characterized by recurrent upper airway collapse, leading to intermittent hypoxia, oxidative stress, and systemic inflammation. Hypoxia-inducible factor-1α (HIF-1α) plays a central role in cellular adaptation to hypoxia, whereas Netrin family members have emerged as regulators of inflammatory and endothelial responses. However, the roles of Netrin-1 and Netrin-4 in OSA-related intermittent hypoxia remain unclear. This study aimed to investigate circulating HIF-1α, Netrin-1, and Netrin-4 levels in patients with OSA and to evaluate their associations with disease severity and hypoxic burden. ***Materials and Methods*:** This study included 52 patients with newly diagnosed OSA and 26 healthy controls. Participants were classified as severe OSA, mild-moderate OSA, or control according to apnea–hypopnea index (AHI) values. All participants underwent overnight polysomnography. Serum HIF-1α, Netrin-1, and Netrin-4 levels were measured using ELISA. ROC curve analyses were performed to assess the ability of the investigated biomarkers to distinguish patients with OSA from controls. Correlation analyses evaluated associations between biomarkers and polysomnographic parameters, while multiple linear regression analyses adjusted for BMI, age, gender, CRP, and LDL levels were used to identify independent associations. ***Results*:** Serum HIF-1α, Netrin-1, and Netrin-4 levels differed significantly among the groups (*p* < 0.0001) and progressively increased from controls to mild-moderate and severe OSA groups. ROC curve analyses demonstrated excellent discriminative performance for HIF-1α (AUC = 0.9072) and Netrin-1 (AUC = 0.8928), while Netrin-4 also showed good discriminative ability (AUC = 0.8284). HIF-1α levels were positively correlated with both AHI, reflecting disease severity, and T90, reflecting nocturnal hypoxic burden (*p* < 0.0001). Similar positive correlations were observed between Netrin-1, Netrin-4, and both AHI and T90 (*p* < 0.0001). In multiple linear regression analyses, AHI remained independently associated with HIF-1α, Netrin-1, and Netrin-4 after adjustment for BMI, age, gender, CRP, and LDL levels (*p* < 0.0001). In contrast, BMI, age, gender, CRP, and LDL were not significantly associated with any of these biomarker levels in the adjusted models. ***Conclusions*:** Circulating HIF-1α, Netrin-1, and Netrin-4 levels are significantly elevated in patients with OSA and are positively associated with disease severity and hypoxic burden. The independent relationship between AHI and these biomarkers suggests that intermittent hypoxia may contribute to activation of HIF-1α–related and Netrin-associated pathways in OSA. These findings indicate that Netrin family members may have potential relevance as biomarkers of hypoxia-associated inflammatory and endothelial alterations in OSA.

## 1. Introduction

Obstructive sleep apnea (OSA) is a disorder characterized by repetitive collapse of the upper airway during sleep, leading to intermittent oxygen desaturations and disrupted sleep architecture [1]. It is a highly prevalent condition, affecting an estimated 5–15% of the general adult population and increasing in frequency with age [2]. OSA represents a significant public health problem. However, it remains markedly underdiagnosed, and approximately 80–90% of individuals with moderate to severe OSA are believed to be unrecognized and untreated in clinical practice [3]. This low diagnosis rate may leave many patients untreated, thereby contributing to impaired quality of life and increased risks of cardiovascular and metabolic complications [4]. Beyond its effects on sleep quality, OSA has major clinical relevance because of its close association with cardiovascular and metabolic comorbidities, including hypertension, coronary artery disease, arrhythmias, stroke, and metabolic dysfunction [5].

Intermittent hypoxemia due to repeated apneic events is now recognized as a key driver of OSA’s systemic consequences, contributing to cardiovascular and metabolic complications and even potentially to cancer progression [6]. Importantly, intermittent hypoxia in OSA is characterized by repetitive cycles of hypoxia-reoxygenation, which have been shown to promote oxidative stress and contribute to systemic inflammation [7,8]. Hypoxia itself acts as a potent inflammatory stimulus by activating transcription factors such as nuclear factor-kappa B (NF-κB), which in turn upregulates the expression of proinflammatory genes [9,10]. Indeed, experimental models of OSA’s hallmark intermittent hypoxia demonstrate a systemic inflammatory response characterized by elevated cytokine levels and activation of NF-κB and hypoxia-inducible factor-1α (HIF-1α) pathways [11]. These pathways are implicated in OSA pathophysiology and may contribute to chronic low-grade inflammation and cardiometabolic complications [12,13]. Mucosal organs with large surface areas, such as the lungs and intestines, are particularly prone to hypoxia-induced inflammation [14]. Given that hypoxia and inflammation are closely linked processes, particularly at mucosal surfaces, local hypoxia-induced inflammatory responses may contribute to relevant pathophysiological mechanisms [15]. At the same time, hypoxia triggers counter-regulatory mechanisms, and HIF-1α activation can coordinate anti-inflammatory and tissue-protective responses that help limit damage caused by low-oxygen conditions [16].

Netrins are a family of extracellular, laminin-related proteins that were first identified as guidance cues directing cell migration and axon pathfinding during neural development, with Netrin-1 being the most extensively studied member. Netrin-1 exerts its effects via several receptors, including the Deleted in Colorectal Cancer (DCC) receptor, its homolog neogenin, and Unc-5 Netrin receptor B (UNC5B) [17]. Beyond its developmental guidance role, Netrin-1 has emerged as an important immunomodulatory molecule that can attenuate inflammation by inhibiting the recruitment and transmigration of leukocytes into tissues. For example, Netrin-1 has been shown to block monocyte adhesion and migration by suppressing NF-κB–mediated expression of chemokines and adhesion molecules, thereby reducing the production of proinflammatory cytokines [18]. Consistently, in hypoxic inflammatory conditions, HIF-1α–driven expression of Netrin-1 in mucosal tissues helps dampen inflammation; Netrin-1 can limit the infiltration of neutrophils during hypoxia-induced inflammation [14]. Notably, dysregulated Netrin-1 levels have been observed in several metabolic and inflammatory diseases and circulating Netrin-1 has been reported to be altered in obesity and type 2 diabetes, suggesting that Netrin-1 may serve as a biomarker or therapeutic target in these conditions [19]. Netrin-1 has been shown to inhibit leukocyte migration and dampen hypoxia-associated inflammatory responses [20,21]. In OSA, where intermittent hypoxia is associated with inflammatory activation, these properties suggest that Netrin-1 may represent a potential endogenous modulator of inflammation. Netrin-4 is another secreted member of the Netrin family, originally termed β-Netrin, and is involved in axon guidance, cell migration, basement membrane organization, angiogenesis, and endothelial homeostasis [22]. Emerging evidence suggests that Netrin-4 may exert protective effects on endothelial cells by attenuating inflammatory activation and preserving endothelial homeostasis [23]. Netrin-4 is highly enriched in vascular basement membranes and has been associated with vascular maturation and stabilization during angiogenesis [22,24]. In addition, experimental studies have shown that Netrin-4 expression may be influenced by hypoxic conditions [24]. Given the role of endothelial dysfunction and chronic intermittent hypoxia in OSA-related complications, these findings suggest that Netrin-4 may have potential relevance in OSA-associated vascular and inflammatory alterations.

Taken together, these findings suggest that Netrin family members may play a critical role in modulating the balance between pro-inflammatory and protective responses under hypoxic conditions. However, despite growing evidence regarding the involvement of Netrin-1 and Netrin-4 in inflammation and vascular biology, their potential roles in OSA-associated intermittent hypoxia remain poorly understood. Evaluating these molecules may provide further insight into the inflammatory and vascular mechanisms associated with OSA and may help clarify their potential clinical relevance. Therefore, the present study aimed to investigate the circulating levels of Netrin-1 and Netrin-4 in patients with OSA and to evaluate their potential associations with clinical and biochemical markers of disease severity and hypoxic burden.

## 2. Material and Methods

### 2.1. Study Design and Population

**Figure 1:**
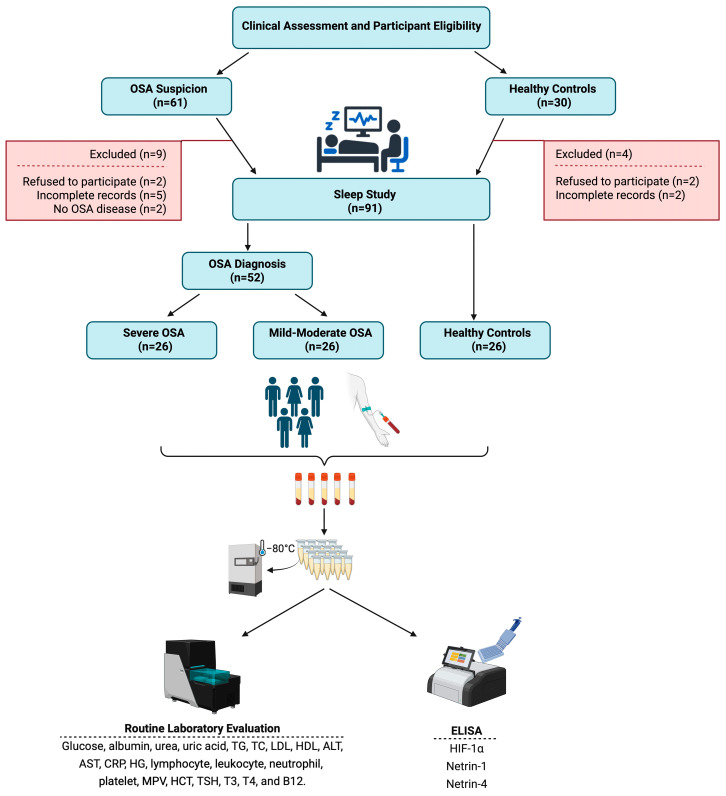
Flow diagram of participant selection, group allocation, blood sample collection, and subsequent laboratory and ELISA analyses in the study population. OSA, obstructive sleep apnea; TG, triglycerides; TC, total cholesterol; LDL, low-density lipoprotein; HDL, high-density lipoprotein; ALT, alanine aminotransferase; AST, aspartate aminotransferase; CRP, C-reactive protein; HG, hemoglobin; MPV, mean platelet volume; HCT, hematocrit; TSH, thyroid-stimulating hormone; T3, triiodothyronine; T4, thyroxine; B12, vitamin B12. Created in BioRender. Erdem, Ş. (2026) https://BioRender.com/m6pzyge, accessed on 3 July 2026.

This observational study was conducted between September 2024 and March 2025 at the Department of Pulmonary Medicine, Faculty of Medicine, Malatya Turgut Özal University. The study population comprised individuals who were newly evaluated for OSA. A total of 52 patients diagnosed with OSA were enrolled and categorized according to disease severity based on established clinical and polysomnographic criteria. Of these, 26 patients were classified as having severe OSA, while 26 were assigned to the mild-to-moderate OSA group. In addition, a control group consisting of 26 healthy subjects was included. These individuals showed no clinical or laboratory findings suggestive of OSA or any other sleep disorder. To reduce potential confounding effects, efforts were made to achieve comparable distributions of age, gender, and body mass index (BMI) between the control and patient groups. Participants aged 20 years and older were considered eligible. Exclusion criteria included current or past smoking, a history of malignancy, prior diagnosis or treatment of OSA, the presence of any other sleep disorder, and any acute or chronic condition such as cardiovascular or cerebrovascular ischemic disease, chronic obstructive pulmonary disease (COPD), active infection, renal impairment, thyroid dysfunction, or chronic inflammatory disease. Individuals receiving regular medications or herbal supplements with potential effects on inflammatory or metabolic pathways were also excluded. All participants underwent a standardized assessment prior to enrollment. The sample size was determined by power analysis using G*Power software version 3.1. Based on an effect size of 0.46, a significance level of 0.05, and a statistical power of 95%, the minimum required sample size was calculated as 78 participants, with 26 participants in each group. The participant selection process and group allocation are illustrated in a flow diagram (Figure 1).

### 2.2. Polysomnography

All participants underwent overnight polysomnographic evaluation using a 55-channel digital recording system (Alice 6^®^ Sleepware, Philips Respironics, Murrysville, PA, USA). The recordings included standard physiological signals, including electroencephalography, electrooculography, electromyography, airflow, respiratory effort, and oxygen saturation. All raw data were scored and interpreted by experienced clinicians in accordance with the criteria established by the American Academy of Sleep Medicine (AASM), specifically following the AASM scoring manual version 2.4 [25]. Respiratory events were defined using standardized thresholds. Apnea was identified as a reduction of at least 90% in peak thermal sensor excursion from baseline, lasting for a minimum duration of 10 s. Hypopnea was defined as a decrease of at least 30% in the nasal pressure signal relative to baseline, persisting for at least 10 s, and accompanied by either a minimum 3% decline in oxygen saturation (SaO_2_) from the pre-event baseline or an arousal from sleep. Oxygen desaturation events were defined as a decrease of 3% or more in oxygen saturation compared with pre-event baseline levels. Several oxygenation parameters were also derived from the recordings. The minimum SaO_2_ was defined as the lowest value recorded during the entire sleep period, whereas the mean SaO_2_ represented the average oxygen saturation throughout the night. T90 was defined as the percentage of total sleep time spent with oxygen saturation below 90% and was evaluated as an indicator of cumulative nocturnal hypoxic burden. The apnea-hypopnea index (AHI), a key metric in OSA assessment, was calculated as the mean number of apneic and hypopneic events per hour of sleep. An AHI value greater than 5 events per hour was accepted as indicative of obstructive sleep apnea. Based on AHI severity classification, patients were stratified into two groups: those with mild-moderate OSA, defined by an AHI of 5 ≤ AHI < 30 events per hour, and those with severe OSA, defined by an AHI of 30 events per hour or higher. Individuals with an AHI below 5 events per hour were categorized as the control group in accordance with AASM recommendations [26].

### 2.3. Sample Collection and Routine Laboratory Evaluation

Venous blood samples were obtained from the brachial vein of both patients and healthy controls between 08:00 and 09:00 a.m., immediately following overnight polysomnography. Blood was collected into gel separator tubes for serum analyses and into ethylenediaminetetraacetic acid (EDTA) tubes for complete blood count measurements. All tubes were transported to the laboratory under appropriate conditions. Serum tubes were allowed to clot for approximately 20–30 min and subsequently centrifuged at 1800× *g* for 10 min at room temperature. The separated serum was used for biochemical measurements, including glucose, albumin, urea, uric acid, triglycerides, total cholesterol, low-density lipoprotein (LDL), high-density lipoprotein (HDL), alanine aminotransferase (ALT), aspartate aminotransferase (AST), and C-reactive protein (CRP), as well as for hormone analyses, including thyroid-stimulating hormone (TSH), triiodothyronine (T3), thyroxine (T4), and vitamin B12. Biochemical parameters were analyzed using an Abbott Architect c16000, whereas hormone assays were performed with a Roche Diagnostics Cobas e601. Complete blood count parameters, including hemoglobin, lymphocyte, leukocyte, neutrophil, platelet, mean platelet volume (MPV), and hematocrit (HCT) were determined from EDTA-anticoagulated samples using a Sysmex XN-1000. The remaining serum fractions were aliquoted into micro-volume tubes (1.5 mL) and stored at −80 °C until further analyses.

### 2.4. Measurement of Serum HIF-1α, Netrin-1, and Netrin-4 Levels

Serum levels of HIF-1α, Netrin-1, and Netrin-4 were measured in stored serum samples on the day of analysis using commercially available enzyme-linked immunosorbent assay (ELISA) kits. HIF-1α (Bioassay Technology Laboratory, Cat. No: EA00069Hu, Jiaxing, China), Netrin-1 (ELK Biotechnology, Catalog Number: ELK2210, Sugar Land, TX, USA), and Netrin-4 (ELK Biotechnology, Catalog Number: ELK2211, Sugar Land, TX, USA) were analyzed according to the manufacturers’ protocols. The detection ranges were 0.1–38 ng/mL for HIF-1α, 31.25–2000 pg/mL for Netrin-1, and 7.82–500 pg/mL for Netrin-4. The results were expressed in ng/mL for HIF-1α and pg/mL for Netrin-1 and Netrin-4.

### 2.5. Statistical Analysis

All statistical analyses were performed using GraphPad Prism (Version 11.0.1 (99); GraphPad Software, San Diego, CA, USA). The distribution of continuous variables was assessed using the Shapiro–Wilk test. As most variables did not demonstrate normal distribution, non-parametric methods were used for group comparisons. Continuous variables are presented as a median (interquartile range, IQR), whereas categorical variables are expressed as a number (percentage). Comparisons among the three study groups were performed using the Kruskal–Wallis test, followed by Dunn’s multiple comparisons test with Bonferroni-adjusted *p*-values for post hoc pairwise analyses when appropriate. Receiver operating characteristic (ROC) curve analysis was performed to evaluate the diagnostic performance of the investigated biomarkers, and the area under the curve (AUC), sensitivity, specificity, and optimal cut-off values were calculated. Correlations were assessed using Spearman’s rank correlation coefficient (r), and a correlation matrix was constructed to visualize the direction and strength of the relationships among demographic, metabolic, inflammatory, polysomnographic, and biomarker variables. Multiple linear regression analyses were conducted after assessment of model assumptions to determine the independent associations between biomarker levels and AHI after adjustment for potential confounding variables. Categorical variables were compared using the chi-square test. A two-tailed *p*-value < 0.05 was considered statistically significant.

## 3. Results

### 3.1. Baseline Demographic Characteristics

In the overall cohort, 36 participants (46.2%) were female and 42 (53.8%) were male. Gender distribution was comparable among the severe OSA, mild–moderate OSA, and control groups (*p* = 0.856). Age also did not differ significantly across the groups (*p* = 0.232), indicating a similar age distribution. In contrast, BMI showed a significant group difference (*p* < 0.0001). Pairwise comparisons demonstrated that BMI was higher in both the severe and mild–moderate OSA groups than in the control group, whereas BMI values were similar between the severe and mild-moderate OSA groups (Table 1).

### 3.2. Polysomnographic Findings

AHI values differed markedly among the groups (*p* < 0.0001), demonstrating a progressive increase from the control group to the mild–moderate OSA group and reaching the highest levels in the severe OSA group. All intergroup comparisons for AHI were statistically significant. Minimum oxygen saturation levels also varied significantly across the groups (*p* < 0.0001). The severe OSA group exhibited the lowest minimum saturation values, followed by the mild–moderate OSA group, whereas the control group showed the highest levels. Significant differences were observed between all groups. Mean oxygen saturation likewise differed significantly among the groups (*p* < 0.0001). The severe OSA group had lower mean oxygen saturation levels than both the mild–moderate OSA and control groups, while no significant difference was detected between the mild–moderate OSA and control groups. T90 values demonstrated a significant stepwise increase across the groups (*p* < 0.0001), with the highest values observed in the severe OSA group and the lowest values in controls. All intergroup comparisons for T90 were statistically significant (Table 2).

### 3.3. Routine Laboratory Findings

Glucose, albumin, urea, uric acid, triglycerides, total cholesterol, HDL, ALT, AST, hemoglobin, lymphocyte, leukocyte, neutrophil, platelet, MPV, hematocrit, TSH, T3, T4, and vitamin B12 levels were comparable among the groups (*p* > 0.05). In contrast, LDL levels differed significantly across the groups (*p* = 0.009), with higher levels observed in the severe OSA group compared to controls, whereas the remaining intergroup comparisons were not significant. CRP levels also showed a significant group difference (*p* = 0.03), primarily driven by elevated CRP levels in the severe OSA group relative to the control group (Table 3).

### 3.4. Serum HIF-1α, Netrin-1, and Netrin-4 Levels

**Figure 2:**
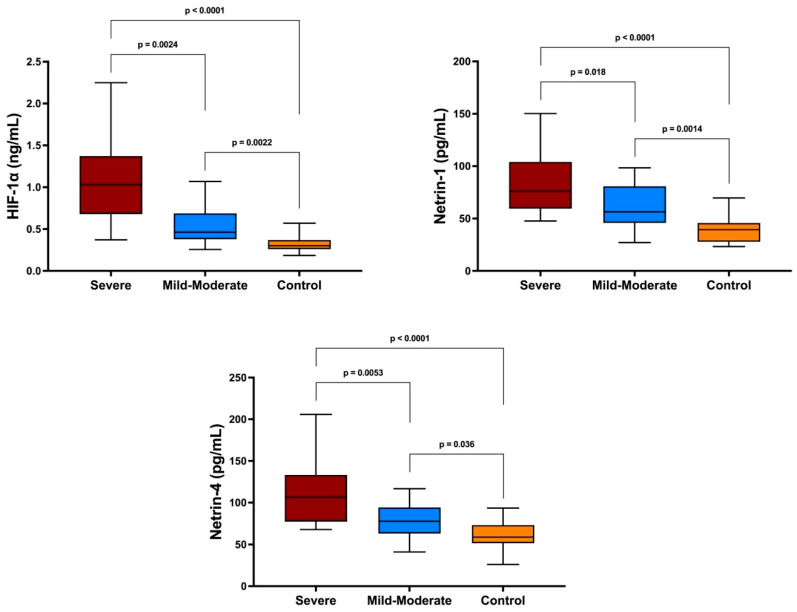
Comparison of serum HIF-1α, Netrin-1, and Netrin-4 levels among patients with severe OSA, mild–moderate OSA, and healthy controls. Data are presented as box-and-whisker plots showing median, interquartile range, and minimum–maximum values. Statistically significant pairwise comparisons are indicated in the figure.

HIF-1α levels demonstrated a progressive increase across the groups, with median values of 0.300 in the control group, 0.462 in the mild-moderate OSA group, and 1.030 in the severe OSA group (*p* < 0.0001). HIF-1α levels were significantly higher in the severe OSA group than in both the mild-moderate OSA and control groups, while the mild-moderate OSA group also exhibited higher levels compared to controls. A comparable distribution pattern was observed for Netrin-1. Median Netrin-1 levels were 39.36 in the control group, 56.41 in the mild–moderate OSA group, and 76.04 in the severe OSA group (*p* < 0.0001). Both OSA groups demonstrated higher Netrin-1 levels than controls, and the severe OSA group additionally showed higher levels than the mild–moderate OSA group. Similarly, Netrin-4 levels increased in parallel with disease severity, with median values of 58.92, 77.92, and 106.4 in the control, mild–moderate OSA, and severe OSA groups, respectively (*p* < 0.0001). Netrin-4 levels were significantly elevated in the severe OSA group compared with both the mild–moderate OSA and control groups, and the mild–moderate OSA group also had higher levels than the control group (Figure 2).

### 3.5. ROC Curve Analyses

**Figure 3:**
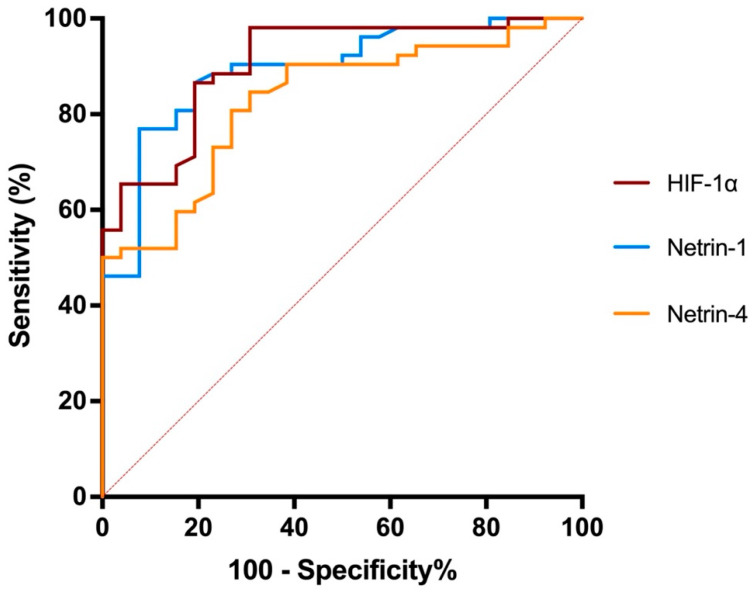
ROC curve analysis of HIF-1α, Netrin-1, and Netrin-4 for discriminating patients with obstructive sleep apnea from healthy controls. The dashed diagonal line indicates the line of no discrimination (AUC = 0.5).

ROC curve analyses were performed to evaluate the discriminative ability of HIF-1α, Netrin-1, and Netrin-4 in distinguishing patients with OSA from healthy controls. HIF-1α demonstrated excellent diagnostic performance, with an area under the curve (AUC) of 0.9072 (95% CI: 0.8404–0.9740, *p* < 0.0001). Similarly, Netrin-1 also showed excellent discriminatory ability, yielding an AUC of 0.8928 (95% CI: 0.8195–0.9660, *p* < 0.0001). Netrin-4 demonstrated good diagnostic performance with an AUC of 0.8284 (95% CI: 0.7370–0.9199, *p* < 0.0001). Overall, these findings indicate that all three biomarkers possess significant discriminative capacity for identifying OSA patients, with HIF-1α showing the highest overall diagnostic accuracy among the investigated biomarkers (Figure 3).

### 3.6. Correlation Matrix Analysis of Clinical, Polysomnographic, and Biomarker Variables

A Spearman correlation matrix was constructed to provide an integrated overview of the relationships among demographic, metabolic, inflammatory, polysomnographic, and biomarker variables. AHI showed significant positive correlations with HIF-1α (r = 0.5460, 95% CI: 0.3683–0.6853, *p* < 0.0001), Netrin-1 (r = 0.5201, 95% CI: 0.3365–0.6656, *p* < 0.0001), and Netrin-4 (r = 0.5977, 95% CI: 0.4327–0.7239, *p* < 0.0001). Among the investigated biomarkers, Netrin-4 showed the strongest positive correlation with AHI.

**Figure 4:**
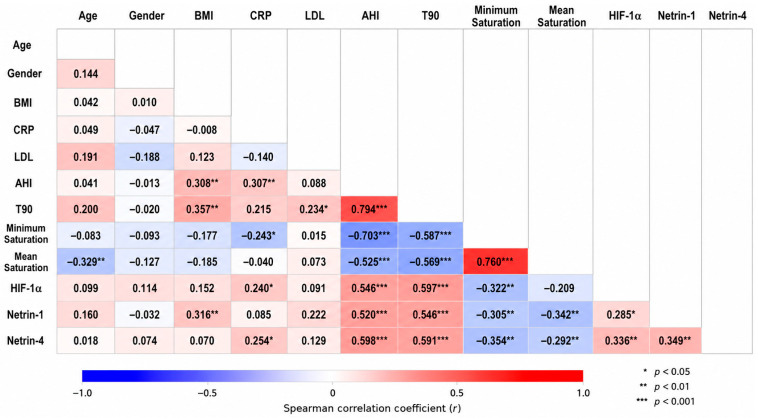
Spearman correlation matrix of demographic, metabolic, inflammatory, polysomnographic, and biomarker variables. The heatmap displays Spearman correlation coefficients (r) among age, gender, BMI, CRP, LDL, AHI, T90, minimum oxygen saturation, mean oxygen saturation, HIF-1α, Netrin-1, and Netrin-4. Positive correlations are shown in red and negative correlations in blue, with color intensity reflecting the magnitude of the correlation coefficient. Gender was coded as 0 = male and 1 = female. Asterisks indicate statistically significant correlations: * *p* < 0.05, ** *p* < 0.01, and *** *p* < 0.001.

Similarly, T90 showed significant positive correlations with HIF-1α (r = 0.5967, 95% CI: 0.4314–0.7232, *p* < 0.0001), Netrin-1 (r = 0.5463, 95% CI: 0.3686–0.6855, *p* < 0.0001), and Netrin-4 (r = 0.5909, 95% CI: 0.4241–0.7189, *p* < 0.0001). Direct comparison of the correlation coefficients showed that T90 was numerically more strongly correlated with HIF-1α (r = 0.597 vs. 0.546) and Netrin-1 (r = 0.546 vs. 0.520) than AHI, whereas AHI showed a slightly stronger correlation with Netrin-4 (r = 0.598 vs. 0.591). Overall, AHI and T90 demonstrated broadly comparable associations with the investigated biomarkers, with no consistent superiority of one OSA severity measure over the other (Figure 4).

### 3.7. Multiple Linear Regression Analyses of Biomarker Levels

In multiple linear regression analyses adjusted for BMI, age, gender, CRP, and LDL levels, AHI remained independently associated with HIF-1α levels (β = 0.0082, 95% CI: 0.0053–0.0110, *p* < 0.0001). None of the covariates, including BMI (*p* = 0.9214), age (*p* = 0.3765), gender (*p* = 0.3186), CRP (*p* = 0.3603), or LDL (*p* = 0.4689), showed a significant association with HIF-1α levels. The overall model was statistically significant (F(6, 71) = 8.077, *p* < 0.0001) and explained 40.6% of the variance in HIF-1α levels (R^2^ = 0.4057). Similarly, AHI remained independently associated with Netrin-1 levels (β = 0.3896, 95% CI: 0.2119–0.5673, *p* < 0.0001), whereas BMI (*p* = 0.1504), age (*p* = 0.2544), gender (*p* = 0.8262), CRP (*p* = 0.6489), and LDL (*p* = 0.2154) were not significant contributors. The overall model was statistically significant (F(6, 71) = 6.012, *p* < 0.0001) and explained 33.7% of the variance (R^2^ = 0.3369). For Netrin-4, AHI also remained independently associated with serum Netrin-4 levels (β = 0.5882, 95% CI: 0.3872–0.7892, *p* < 0.0001), while no significant associations were observed for BMI (*p* = 0.1875), age (*p* = 0.6061), gender (*p* = 0.2107), CRP (*p* = 0.3365), or LDL (*p* = 0.1681). The overall model was statistically significant (F(6, 71) = 7.874, *p* < 0.0001) and explained 40.0% of the variance (R^2^ = 0.3995). Collectively, these findings demonstrate that the associations between AHI and HIF-1α, Netrin-1, and Netrin-4 levels remained significant after additional adjustment for BMI, age, gender, CRP, and LDL (Table 4).

## 4. Discussion

The present study demonstrated that circulating HIF-1α, Netrin-1, and Netrin-4 levels were significantly elevated in patients with OSA and increased progressively according to disease severity. In addition to marked intergroup differences, all three biomarkers showed significant positive correlations with both AHI, reflecting disease severity, and T90, reflecting nocturnal hypoxic burden, suggesting a close relationship between OSA severity, cumulative hypoxic exposure, and activation of hypoxia-related inflammatory pathways. Moreover, ROC curve analyses revealed excellent diagnostic performance for HIF-1α and Netrin-1 and good discriminative ability for Netrin-4, while multiple linear regression analyses confirmed that AHI remained independently associated with all investigated biomarkers after adjustment for BMI, age, gender, CRP, and LDL levels. Collectively, these findings support a potential relationship between intermittent hypoxia and increased circulating HIF-1α and Netrin-associated biomarker levels in OSA.

Intermittent hypoxia is considered one of the principal mechanisms underlying the systemic consequences of OSA. Recurrent hypoxia–reoxygenation cycles promote oxidative stress, inflammatory signaling, endothelial dysfunction, and sympathetic activation [7,12]. HIF-1α is a master transcription factor activated under hypoxic conditions and regulates multiple adaptive pathways involved in angiogenesis, cellular metabolism, inflammation, and vascular homeostasis [13]. Previous experimental and clinical studies have demonstrated increased HIF-1α activation in OSA, particularly in association with hypoxic burden and disease severity. Increased circulating HIF-1α levels have previously been reported in patients with OSA, particularly in severe disease, and were shown to correlate positively with indices of nocturnal hypoxemia and AHI. In addition, elevated NF-κB and HIF-1α expression levels have been observed in severe OSA patients, further supporting the contribution of intermittent hypoxia to hypoxia-related inflammatory signaling pathways [27,28]. In the present study, HIF-1α levels progressively increased from controls to mild–moderate and severe OSA groups. Moreover, ROC curve analysis demonstrated excellent discriminative performance of HIF-1α for distinguishing patients with OSA from healthy controls. HIF-1α levels also showed significant positive correlations with both AHI and T90. Importantly, AHI remained independently associated with HIF-1α levels after adjustment for BMI, age, gender, CRP, and LDL, suggesting that the observed elevation was not solely attributable to obesity or systemic inflammation but was closely linked to OSA severity itself.

Netrin-1 was initially identified as an axonal guidance molecule but has increasingly been recognized as an important regulator of inflammation and endothelial function [20]. Experimental evidence indicates that hypoxia may directly induce Netrin-1 expression through HIF-1α–dependent transcriptional mechanisms [21]. In mucosal and vascular tissues, Netrin-1 appears to exert anti-inflammatory effects by limiting leukocyte migration and suppressing excessive inflammatory activation [20,21]. HIF-1α–dependent induction of Netrin-1 has been shown to attenuate hypoxia-induced inflammation by reducing neutrophil transmigration through adenosine receptor–mediated mechanisms. These observations are particularly relevant in OSA, where recurrent hypoxia contributes to chronic low-grade inflammation and endothelial dysfunction [14,21]. Our findings demonstrated a stepwise increase in Netrin-1 levels parallel to OSA severity. Moreover, ROC curve analysis demonstrated excellent discriminative performance of Netrin-1 for distinguishing patients with OSA from healthy controls. Netrin-1 levels also showed significant positive correlations with both AHI and T90. Furthermore, AHI remained independently associated with Netrin-1 levels in adjusted regression analyses. Together, the associations of Netrin-1 with both AHI and T90, along with its independent association with AHI in adjusted models, suggest that circulating Netrin-1 levels may be related to both OSA severity and nocturnal hypoxic exposure. Interestingly, Netrin-1 has been reported to exhibit dual behavior depending on disease context. Increased Netrin-1 expression has been suggested to represent a compensatory response aimed at limiting hypoxia-induced inflammatory injury at mucosal surfaces [21]. However, persistently elevated circulating Netrin-1 levels have also been reported in chronic inflammatory and metabolic disorders associated with endothelial dysfunction [19,29].

Netrin-4 is involved in basement membrane organization, endothelial integrity, angiogenesis, and vascular remodeling [22]. Emerging studies suggest that Netrin-4 may protect endothelial cells from inflammatory injury and contribute to maintenance of vascular homeostasis [23]. Because endothelial dysfunction is a central component of OSA-related cardiovascular complications, alterations in Netrin-4 may have important pathophysiological implications. In the present study, Netrin-4 levels demonstrated a progressive increase according to disease severity. Moreover, ROC curve analysis showed good discriminative performance of Netrin-4 for distinguishing patients with OSA from healthy controls. Netrin-4 levels were also positively correlated with both AHI and T90. Similar to HIF-1α and Netrin-1, AHI remained independently associated with Netrin-4 levels after adjustment for potential confounders. These findings support a potential association between Netrin-4 and hypoxia-related alterations observed in OSA. Although direct evidence regarding Netrin-4 in OSA is currently limited, the present results support the hypothesis that Netrin family members may be associated with hypoxia-related endothelial and inflammatory alterations in OSA.

One of the notable aspects of the present study was the ROC curve analysis evaluating the exploratory biomarker performance of the investigated biomarkers. HIF-1α demonstrated strong discriminative performance, with excellent ability to distinguish OSA patients from controls (AUC > 0.90), while Netrin-1 also showed excellent performance and Netrin-4 demonstrated good diagnostic accuracy. These findings suggest that hypoxia-related biomarkers may have potential clinical relevance in identifying patients with OSA. Although polysomnography remains the gold standard for diagnosis, its costly and time-consuming nature, requirement for specialized personnel, and limited availability continue to restrict widespread screening [30]. Therefore, circulating biomarkers associated with intermittent hypoxia may provide supportive value in identifying individuals at increased risk for OSA or in reflecting disease severity. Nevertheless, these findings should be interpreted cautiously because biomarker-based diagnostic strategies cannot currently replace polysomnographic evaluation. The correlation analyses further strengthened the biological plausibility of the observed findings. Both AHI and T90 showed moderate positive correlations with HIF-1α, Netrin-1, and Netrin-4. Direct comparison of the correlation coefficients showed that T90 was numerically more strongly correlated with HIF-1α and Netrin-1, whereas AHI showed a slightly stronger correlation with Netrin-4. The broadly comparable associations of AHI and T90 with the investigated biomarkers, without consistent superiority of either measure, suggest that event frequency and cumulative hypoxic burden may reflect complementary dimensions of OSA-related biomarker alterations. This observation is important because increasing evidence indicates that hypoxic burden parameters may better reflect the biological consequences of OSA than AHI alone. Recent studies have suggested that indices reflecting oxygen desaturation severity and duration may correlate more strongly with cardiovascular and inflammatory outcomes than conventional event-based metrics [31,32]. Therefore, the significant associations observed between T90 and all investigated biomarkers may further support the central role of intermittent hypoxia in OSA pathophysiology. Another important finding was that BMI, age, gender, CRP, and LDL levels were not independently associated with HIF-1α, Netrin-1, or Netrin-4 levels in multivariable models. Since obesity itself is known to contribute to systemic inflammation and hypoxia-related signaling, it was particularly important to determine whether biomarker elevations were independently linked to OSA severity. Although efforts were made to minimize major BMI differences during participant selection, BMI differed significantly among the study groups, with higher BMI values observed in the OSA groups compared with controls. Given the established relationship between obesity, systemic inflammation, and hypoxia-related signaling pathways, residual confounding related to adiposity cannot be completely excluded. To reduce the potential influence of measured confounding factors, BMI, age, gender, CRP, and LDL were included as covariates in all multivariable regression analyses. Importantly, after adjustment for these variables, AHI remained independently associated with HIF-1α, Netrin-1, and Netrin-4 levels. Nevertheless, future studies using strictly BMI-matched cohorts and more comprehensive adiposity-related assessments, including visceral adiposity and metabolic profiling, are needed to further validate these findings. The persistence of significant associations between AHI and biomarker levels after adjustment suggests that intermittent hypoxia may exert independent effects beyond the contribution of obesity-related inflammation. This finding strengthens the interpretation that the observed biomarker alterations are closely connected to OSA-specific pathophysiological mechanisms.

Several limitations of this study should be acknowledged. First, the cross-sectional and single-center design limits the ability to establish causal relationships and may affect generalizability. Second, the sample size was relatively modest, although statistically significant differences were consistently observed across analyses. Third, only circulating biomarker levels were evaluated. Therefore, tissue-level expression and mechanistic signaling pathways could not be directly assessed. In addition, circulating HIF-1α measurements may be influenced by preanalytical variability, the relatively short half-life of HIF-1α, and potential assay-related limitations associated with commercially available ELISA kits. Therefore, circulating HIF-1α levels may not fully reflect intracellular HIF-1α activity at the tissue level. Additionally, inflammatory cytokines and endothelial function markers beyond CRP were not comprehensively investigated. Finally, although ROC analyses demonstrated promising discriminative ability, external validation in larger and independent cohorts is necessary before clinical applicability can be considered. Despite these limitations, the present study has several strengths. To our knowledge, this is among the first studies simultaneously evaluating HIF-1α, Netrin-1, and Netrin-4 in OSA while comprehensively integrating polysomnographic severity indices, ROC analyses, correlation analyses, and adjusted regression models. The consistent associations observed across disease severity groups, hypoxic burden parameters, and adjusted analyses further support the potential biological relevance of these biomarkers in OSA-associated intermittent hypoxia.

## 5. Conclusions

The present findings demonstrate that circulating HIF-1α, Netrin-1, and Netrin-4 levels are significantly elevated in OSA and are closely associated with disease severity and nocturnal hypoxic burden. The independent associations between AHI and all investigated biomarkers suggest that intermittent hypoxia may be associated with increased circulating HIF-1α, Netrin-1, and Netrin-4 levels in OSA. These biomarkers may therefore reflect adaptive inflammatory and endothelial responses triggered by recurrent nocturnal hypoxia and could potentially serve as supportive indicators of disease-related hypoxic stress.

## Figures and Tables

**Table 1:** Comparison of demographic and clinical characteristics among the study groups.

Variable	Group	Median (IQR)	H Value	*p*-Value	Post Hoc
Age	Severe ^1^	52.50 (47.50–59.00)	2.918	0.232	NS
	Mild-Moderate ^2^	51.00 (40.75–58.75)			
	Control ^3^	45.50 (36.00–57.75)			
BMI	Severe ^1^	28.30 (24.98–29.90)	20.65	<0.0001	1–3, 2–3
	Mild-Moderate ^2^	27.80 (26.03–29.78)			
	Control ^3^	23.65 (22.35–24.50)			

BMI, body mass index (kg/m^2^); IQR, interquartile range; H value, Kruskal–Wallis test statistic; *p*-value, statistical significance; *p* < 0.05, there is a statistically significant difference between the groups; NS, statistically non-significant; ^1^ = Severe, ^2^ = Mild-Moderate, ^3^ = Control.

**Table 2:** Comparison of polysomnographic parameters among the study groups.

Variable	Group	Median (IQR)	H Value	*p*-Value	Post Hoc
AHI (Events/hour)	Severe ^1^	73.60 (54.95–90.75)	68.47	<0.0001	1–2, 1–3, 2–3
	Mild-Moderate ^2^	21.90 (18.18–27.88)			
	Control ^3^	4.15 (2.30–4.52)			
Minimum saturation (%)	Severe ^1^	73.00 (64.00–79.25)	43.77	<0.0001	1–2, 1–3, 2–3
	Mild-Moderate ^2^	83.00 (81.00–85.00)			
	Control ^3^	87.00 (85.00–90.00)			
Mean saturation (%)	Severe ^1^	89.00 (88.00–91.00)	20.96	<0.0001	1–2, 1–3
	Mild-Moderate ^2^	91.50 (91.00–92.00)			
	Control ^3^	93.00 (91.00–94.00)			
T90 (%)	Severe ^1^	46.15 (33.85–57.88)	64.22	<0.0001	1–2, 1–3, 2–3
	Mild-Moderate ^2^	7.95 (3.45–11.45)			
	Control ^3^	0.60 (0.25–1.15)			

AHI, apnea-hypopnea index; T90, percentage of total sleep time with oxygen saturation < 90%; IQR, interquartile range; H value, Kruskal–Wallis test statistic; *p*-value, statistical significance; *p* < 0.05, there is a statistically significant difference between the groups; ^1^ = Severe, ^2^ = Mild-Moderate, ^3^ = Control.

**Table 3:** Comparison of routine laboratory parameters among the study groups.

Variable	Group	Median (IQR)	H Value	*p*-Value	Post Hoc
Glucose (mg/dL)	Severe ^1^	100.50 (90.25–103.50)	5.49	0.06	NS
	Mild-Moderate ^2^	96.50 (89.75–104.80)			
	Control ^3^	91.50 (85.75–97.00)			
Albumin (g/dL)	Severe ^1^	4.20 (4.10–4.40)	43.77	0.07	NS
	Mild-Moderate ^2^	4.40 (4.20–4.70)			
	Control ^3^	4.20 (4.20–4.62)			
Urea (mg/dL)	Severe ^1^	30.60 (28.28–37.28)	0.87	0.64	NS
	Mild-Moderate ^2^	30.55 (26.08–36.23)			
	Control ^3^	30.40 (24.80–36.70)			
Uric acid (mg/dL)	Severe ^1^	5.20 (4.45–5.80)	1.70	0.42	NS
	Mild-Moderate ^2^	5.05 (4.07–5.90)			
	Control ^3^	4.80 (4.17–5.40)			
Triglycerides (mg/dL)	Severe ^1^	121.50 (102.00–166.50)	2.44	0.29	NS
	Mild-Moderate ^2^	115.50 (101.80–147.30)			
	Control ^3^	109.90 (87.75–144.80)			
Total cholesterol (mg/dL)	Severe ^1^	175.00 (146.00–196.50)	2.57	0.27	NS
	Mild-Moderate ^2^	161.00 (128.30–187.50)			
	Control ^3^	152.00 (129.00–186.80)			
LDL (mg/dL)	Severe ^1^	117.20 (99.45–122.40)	9.29	0.009	1–3
	Mild-Moderate ^2^	104.10 (94.23–114.10)			
	Control ^3^	95.80 (89.75–106.70)			
HDL (mg/dL)	Severe ^1^	47.00 (36.08–59.98)	0.03	0.98	NS
	Mild-Moderate ^2^	45.85 (39.45–58.18)			
	Control ^3^	46.65 (37.40–58.33)			
ALT (U/L)	Severe ^1^	24.50 (18.00–32.50)	2.28	0.31	NS
	Mild-Moderate ^2^	23.00 (18.50–39.25)			
	Control ^3^	21.00 (15.00–28.50)			
AST (U/L)	Severe ^1^	21.50 (17.75–25.50)	0.41	0.81	NS
	Mild-Moderate ^2^	20.50 (16.75–30.25)			
	Control ^3^	20.00 (16.75–25.50)			
CRP (mg/dL)	Severe ^1^	0.35 (0.20–1.02)	6.44	0.03	1–3
	Mild-Moderate ^2^	0.20 (0.20–0.40)			
	Control ^3^	0.20 (0.20–0.32)			
Hemoglobin (g/dL)	Severe ^1^	15.15 (13.98–16.15)	5.96	0.05	NS
	Mild-Moderate ^2^	16.60 (14.80–16.43)			
	Control ^3^	14.25 (13.50–15.70)			
Lymphocyte (10^3^/µL)	Severe ^1^	2.74 (2.37–3.31)	4.53	0.10	NS
	Mild-Moderate ^2^	2.74 (2.15–3.37)			
	Control ^3^	2.27 (1.89–3.33)			
Leukocyte (10^3^/µL)	Severe ^1^	7.62 (6.49–8.95)	0.20	0.90	NS
	Mild-Moderate ^2^	7.74 (6.51–8.69)			
	Control ^3^	7.58 (6.31–9.92)			
Neutrophil (10^3^/µL)	Severe ^1^	3.93 (3.28–4.77)	0.37	0.82	NS
	Mild-Moderate ^2^	3.64 (3.17–4.63)			
	Control ^3^	3.75 (3.31–4.71)			
Platelet (10^3^/µL)	Severe ^1^	239.50 (197.00–286.50)	0.89	0.64	NS
	Mild-Moderate ^2^	259.00 (209.30–307.80)			
	Control ^3^	269.00 (200.00–300.30)			
MPV (fL)	Severe ^1^	10.00 (9.67–10.70)	1.50	0.47	NS
	Mild-Moderate ^2^	10.40 (9.70–11.10)			
	Control ^3^	10.20 (9.90–10.90)			
Hematocrit (%)	Severe ^1^	45.80 (43.55–47.03)	2.36	0.30	NS
	Mild-Moderate ^2^	46.55 (45.38–48.15)			
	Control ^3^	46.40 (43.35–47.23)			
TSH (mU/L)	Severe ^1^	1.69 (1.20–2.65)	1.18	0.55	NS
	Mild-Moderate ^2^	1.51 (1.10–2.01)			
	Control ^3^	1.72 (1.35–2.79)			
T3 (pg/mL)	Severe ^1^	3.13 (2.78–3.66)	0.66	0.71	NS
	Mild-Moderate ^2^	3.15 (2.97–3.43)			
	Control ^3^	3.23 (2.99–3.60)			
T4 (pg/mL)	Severe ^1^	1.20 (1.07–1.44)	5.86	0.05	NS
	Mild-Moderate ^2^	1.40 (1.24–1.60)			
	Control ^3^	1.35 (1.09–1.43)			
Vitamin B12 (pg/dL)	Severe ^1^	331.00 (279.30–480.80)	3.26	0.19	NS
	Mild-Moderate ^2^	325.00 (281.50–451.50)			
	Control ^3^	397.50 (326.80–512.00)			

LDL, low-density lipoprotein; HDL, high-density lipoprotein; ALT, alanine aminotransferase; AST, aspartate aminotransferase; CRP, C-reactive protein; TSH, thyroid-stimulating hormone; T3, triiodothyronine; T4, thyroxine; MPV, mean platelet volume; IQR, interquartile range; H value, Kruskal–Wallis test statistic; *p* value, statistical significance; *p* < 0.05, there is a statistically significant difference between the groups; NS, statistically non-significant; ^1^ = Severe, ^2^ = Mild-Moderate, ^3^ = Control.

**Table 4:** Multiple linear regression analyses of HIF-1α, Netrin-1, and Netrin-4 levels.

Dependent Variable	Independent Variable	β Coefficient	*p*	95% CI	
				Lower Bound	Upper Bound
HIF-1α	AHI	0.0082	<0.0001	0.0053	0.0110
	BMI	−0.0012	0.9214	−0.0264	0.0239
	Age	0.0032	0.3765	−0.0040	0.0106
	Gender	−0.0898	0.3186	−0.2682	0.0885
	CRP	0.0829	0.3603	−0.0966	0.2625
	LDL	0.0016	0.4689	−0.0029	0.0062
Netrin-1	AHI	0.3896	<0.0001	0.2119	0.5673
	BMI	1.136	0.1504	−0.4220	2.694
	Age	0.2625	0.2544	−0.1930	0.7181
	Gender	1.218	0.8262	−9.806	12.24
	CRP	−2.545	0.6489	−13.65	8.555
	LDL	0.1783	0.2154	−0.1061	0.4627
Netrin-4	AHI	0.5882	<0.0001	0.3872	0.7892
	BMI	−1.177	0.1875	−2.939	0.5861
	Age	−0.1339	0.6061	−0.6493	0.3816
	Gender	−7.901	0.2107	−20.37	4.573
	CRP	6.095	0.3365	−6.464	18.65
	LDL	0.2247	0.1681	−0.0970	0.5465

HIF-1α, hypoxia-inducible factor-1α; AHI, apnea–hypopnea index; BMI, body mass index; CRP, C-reactive protein; LDL, low-density lipoprotein; β, unstandardized regression coefficient; CI, confidence interval. Multiple linear regression analyses were adjusted for BMI, age, gender, CRP, and LDL levels. Gender was coded as 0 = male and 1 = female. *p* < 0.05 was considered statistically significant.

## Data Availability

The datasets generated and analyzed during this study are available from the corresponding author on reasonable request.
